# Genomic palaeoparasitology traced the occurrence of *Taenia asiatica* in ancient Iran (Sassanid Empire, 2th cent. CE–6th cent. CE)

**DOI:** 10.1038/s41598-022-10690-2

**Published:** 2022-07-14

**Authors:** Zeynab Askari, Frank Ruehli, Abigail Bouwman, Vahid Shariati, Saied Reza Naddaf, Domenico Otranto, Santiago Mas-Coma, Mostafa Rezaeian, Nicole Boenke, Thomas Stöllner, Abolfazl Aali, Iraj Mobedi, Gholamreza Mowlavi

**Affiliations:** 1 Department of Medical Parasitology and Mycology, School of Public Health, Tehran University of Medical Sciences, Tehran, Iran; 2 Institute of Evolutionary Medicine, Zurich University, Zürich, Switzerland; 3 NIGEB Genome Center, National Institute of Genetic Engineering and Biotechnology, Tehran, Iran; 4 Department of Parasitology, Pasteur Institute of Iran, Tehran, Iran; 5 Department of Veterinary Medicine, University of Bari, Valenzano, Italy; 6 Department of Pathobiology, Faculty of Veterinary Science, Bu-Ali Sina University, Hamedan, Iran; 7 Departamento de Parasitologia, Facultad de Farmacia, Universidad de Valencia, Burjassot, Valencia Spain; 8 Institute for Archaeological Science, Ruhr-University Bochum, Bochum, Germany; 9 German Mining-Museum, Bochum, Germany; 10 Archaeological Museum of Zanjan, Zanjan, Iran; 11 Center for Research of Endemic Parasites of Iran (CREPI), Tehran University of Medical Sciences, Tehran, Iran

**Keywords:** Archaeology, Parasitic infection, Next-generation sequencing, Policy and public health in microbiology

## Abstract

Palaeoparasitology investigates parasitological infections in animals and humans of past distance by examining biological remains. Palaeofaeces (or coprolites) are biological remains that provide valuable information on the disease, diet, and population movements in ancient times. Today, advances in detecting ancient DNA have cast light on dark corners that microscopy could never reach. The archaeological site of the Chehrabad salt mine of Achaemenid (550–330 BC) and Sassanid (third–seventh century AD) provides remains of various biotic and abiotic samples, including animal coprolites, for multidisciplinary studies. In the present work, we investigated coprolites for helminth eggs and larvae by microscopy and traced their biological agents’ DNA by Next Generation Sequencing. Our results revealed various helminths, including *Taenia asiatica*, the species introduced in the 1990s. Implementing advanced modern molecular techniques like NGS gives a paramount view of pathogenic agents in space and time.

## Introduction

Palaeoparasitology elucidates facts such as human behavior, ancient diet, migration patterns, human interactions with animals, and the status of parasitic infections in the distant past by identifying parasites from ancient materials^1–3^. This field of study requires archeologists and geologists to provide samples via a continuous research network. Biological remains, particularly coprolites, serve as exhibitors of the biotic components of the digestive tract, allowing us to recover both host pathogens and intestinal microbiota^4^. It is also possible to study unusual behaviors, like cannibalism, by parasite identification in palaeofaeces^5^.

Furthermore, palaeoparasitology challenges using the terms of emerging and re-emerging diseases and widens our view of pathogenic agents in the matter of space and time. Knowledge of parasitic infections in ancient times would help us document the history of parasites that might have appeared and been eliminated in the distant past^6^. Previously, microscopy detected various helminth eggs and larvae in the rehydrated coprolites but came short of identifying the species due to limited discriminative taxonomical features^7^. With the introduction of ELISA, this method successfully detected *Giardia lamblia* cysts in coprolites but failed to identify *Entamoeba histolytica*^8^. Later, DNA-based methods identified falciparum malaria, visceral leishmaniasis, and Chagas disease in the ancient remains^9^. Development of metagenomics (Next Generation Sequencing) allowed detection of entire ancient protozoa DNA in the biological samples without prior knowledge of their genetic structure^9–11^. The Chehrabad salt mine archeological site is located on the Silk Road trading map in Iran. This ancient trading route might have imported unexpected pathogens into new areas via infected passengers and transporting animals^12,13^.

## Material and methods

### Study area

Chehrabad salt mine used to be exploited in the Achaemenid era (6th to fourth centuries BC), the Sasanian dynasty (224 CE–650 CE), till the middle and late Islamic periods. The archaeological excavation and chance discoveries, during which eight salt mummies and skeletal remains of ancient miners were unearthed, received significant public and scientific attention, leading to multi-disciplinary collaborations between Tehran University of Medical Sciences (TUMS), Bochum, Zurich, and Oxford universities^14^. Meanwhile, palaeoenvironmental investigations using dung layers, plant fibers, and osteological remains identified different animals such as *Ovis aries*, *Capra hircus*, *Bos taurus*, *Sus scrofa*, *Vulpes* sp., *Chiropter* sp., *Rattus* sp., avian, amphibian, and lizard nearby the miners^15^.

### Sampling

**Figure 1:**
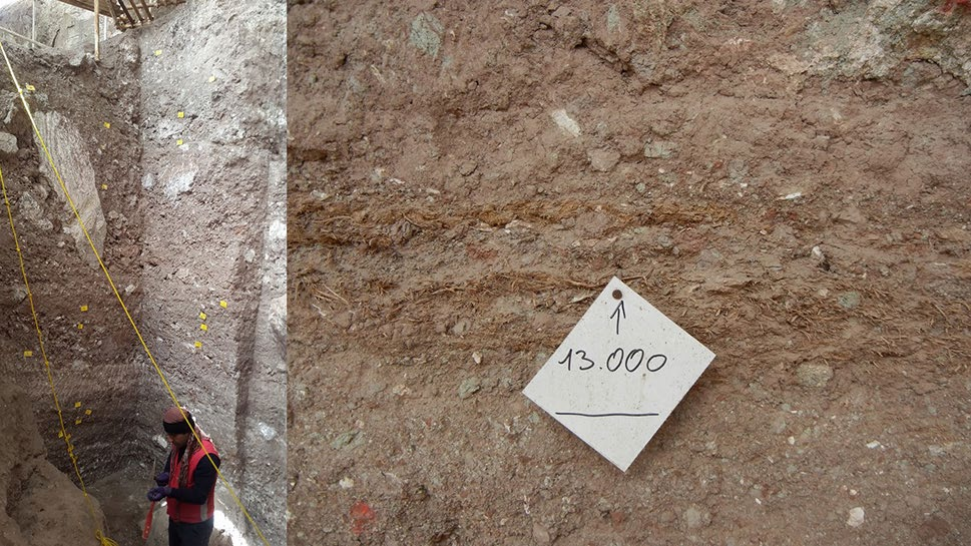
Sampling location: samples were retrieved from vertical layers in 10 to 15 m depth. Edited by Microsoft paint, version 21 H2, https://www.microsoft.com/en-us/windows.

We received 30 palaeofaeces recovered during two rounds of excavations between 2015 and 2017. The samples were obtained from vertical layers in 10–15 m depth (Fig. 1) and immediately photographed and transferred to the labeled plastic bags on the site. The palaeofaeces were dated by stratigraphy and accelerator mass spectrometry as described by others. The origin of faeces was initially determined in Ruhr-University and the German Mining-Museum in Bochum based on morphological features^15^. Carnivore faeces were identified from herbivore and human coprolites by the presence of feathers, fur, or bone fragments (sharp-edged bone fragmentation)^16^. Spiral appearance, reniform pellets, and plant fibers in crushed coprolites were used as macroscopical keys to detect the host for coprolites as bird, donkey, and other herbivores, respectively. The rest of the samples were labeled as unknown origin^17,18^. The samples were divided into two sections for microscopical and molecular studies.

### Microscopy

Five grams of each samples were rehydrated in Tri-Sodium Phosphate (TSP) 0.5% W/V solution for one week at room temperature^19^. Twenty microscopic slides were prepared from two ml of rehydrated material, mounted by glycerin gel, and examined for helminth eggs and larvae by microscopy at 40 X magnification^20^. The eggs and larvae were identified based on morphological features available in taxonomic keys^21^ and photographed using a microscope-equipped camera (LABOMED Lx 500).

### Molecular techniques and analysis

The metagenomic study was carried out on the palaeofaeces in the Institute of Evolutionary Medicine (IEM), Zurich University. The laboratory was equipped exclusively to analyze the biological remains in four separate rooms, i.e., staff changing room, organized sampling section, DNA extraction, and library preparation parts. All facilities were equipped with positive air pressure and UV lamps to prevent modern contamination.

### DNA extraction and library preparation

All samples were first treated with UV for two hours to eliminate any possible modern contamination. A commercial kit (QIAamp Power Fecal DNA Kit) was used to extract DNA, and the quality of the extractions was assessed using a QUBIT fluorometer alongside four blank tubes containing only extraction reagents as negative controls. Libraries were generated from 10 µl of DNA samples using the NEBNext DNA Library Prep Master Set (E6070, New England Biolabs Company), and their quality was checked by the Agilent TapeStation system (United States, California). The libraries were pooled in equimolar concentrations and sequenced by Illumina HiSeq 4000 sequencer (United States) in paired-end mode.

### Bioinformatics analysis

The quality of reads was checked by FastQC software^22^. The reads were screened using Trimmomatic software (version 0.36) set for paired-end mode. Those with "quality and length" ≥ 20 were kept, and the adapters were removed using IlLUMINACLIP option with seedMismatches 2, palindromClip threshold 30, 4-base wide sliding window and simpleClip threshold 10^23^. Using the k-mer alignment method (KMA), all reads were mapped against sequences available at NCBI Nucleotide and Worm-base databases^24^. Low-complex alignments were eliminated, and in the case of reads that mapped to both databases, the ensuing findings were manually evaluated to choose the best hits. Due to the large number of genomic reads assigned to *Cylicostephanus goldi* in four samples, we decided to map those to the given genome separately using the BWA mem algorithm, followed by the GATK4 protocol^25,26^. Sorting coordinate, adding read groups, and removing duplicates were performed using SortSam, AddOrReplaceReadGroup, and MarkDuplicates commands in Picard tools of GATK4 successively. The best alignments were selected using the samtools flag 2304 and 4 to filter the supplementary and secondary aligned reads^27^, and the final filtered alignments were also rescaled by mapDamage computational framework^28^. Following GATK4 protocol, the variant calling format (vcf) and recalibrated binary alignment map (bam) files were created, and single nucleotide polymorphisms (SNPs) and insertion/deletion (INDELs) were calculated using bcftools**.** The best rescaled bam files were merged to create a VCF file using the HaplotypeCaller command in the GATK4. The SNP and INDEL were extracted and filtered using the SelectVariants and VariantFilteretion command with default parameters. Both SNPs and INDELs were further used for recalibration of bam files. VcfR package was used to visualize a scaffold (no.14) with a high variation rate^29^. Since our samples were not too old, the characteristics ancient DNA damage, i.e., cytosine (C) to thymidine (T) transitions at the 5′ ends and guanine (G) to adenosine (A) transitions at the 3' ends of DNA reads, were expected to be less than in 38,000 years old Neanderthal specimens^30^. Also, sodium chloride (NaCl) desiccating effect, which minimizes hydrolysis, results to reduced C to T transitions^31^. Here, these patterns were checked for *C. goldi* reads, the reads with the lesions were separated, and the damage patterns were depicted by PMDtools^32^. The Samtools programs was used to compute the number of reads matched with the *C. goldi* genome in the main bam file (not filtered) and the alignment file comprising only damaged reads**.** In the separate analysis, the ninety reads from *T. asiatica* (from kma output) were separated and aligned to the whole genomes of *T. asiatica*, *Taenia solium*, and *Taenia saginata,* available in worm base database, using the BWA-Mem algorithm for definitive identification. The aligned contigs/scaffolds were selected, then the ninety reads, *T. asiatica* contig (TASK_contig0004697), and *T. saginata* scaffold (Scaffold00355) were realigned with *T. solium* contig (pathogen_TSM_contig_01480). The same procedure was done using the assembled or concatenated sequence of ninety reads in KMA output (fsa file), and *T. saginata* scaffold (Scaffold00355) was selected as the reference. The network of possible interactions was also drawn using igraph^33^.

## Results

### Dating and sample origin

Eight coprolites among the samples belonged to the Achaemenid time (6th cent. BCE–3th cent. BCE) and twenty-two to the Sassanid era (2th cent. CE-6th cent. CE). Using visible features and microscopical images, six donkeys, seven herbivores, and two birds were identified as the samples' origins. The remaining fifteen coprolites were labeled as samples of unknown origin (Supplementary file result).

### Parasite detection

**Figure 2:**
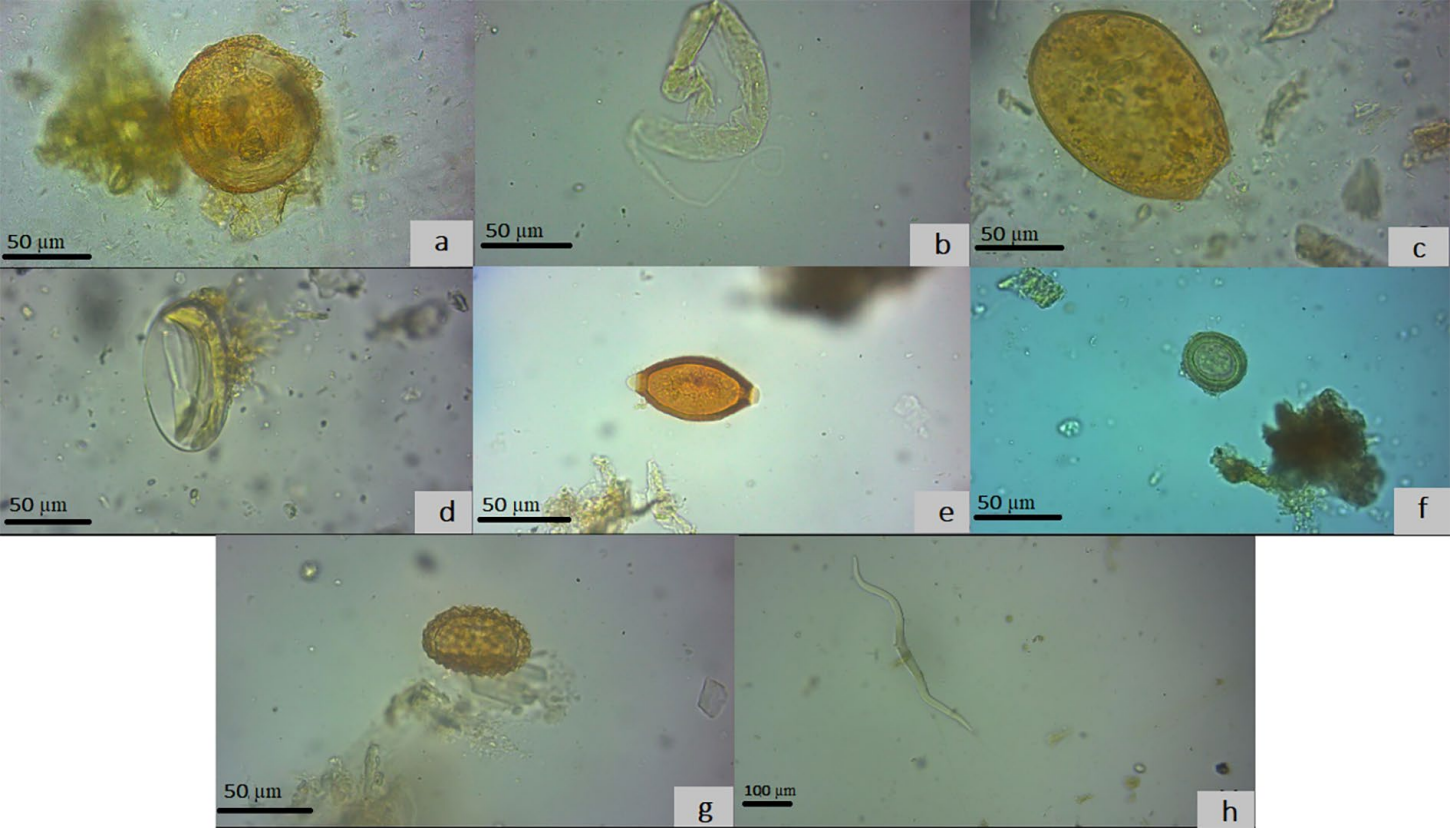
The microscopic results: From top left to right: (**a**): *Parascaris* sp. egg, (**b**): Strongyle larvae, (**c**): *Fasciola* sp. egg, (**d**): Strongyle egg, (**e**): *Trichuris *spp. egg, (**f**): *Taenia* spp. egg, (**g**): *Ascaris* sp. egg, and (**h**): unknown nematode larvae. Edited by Microsoft paint, version 21 H2, https://www.microsoft.com/en-us/windows.

Examining one thousand microscopic slides from the rehydrated palaeofaeces revealed seven different helminth species belonging to *Ascaris* sp., strongyles, Trichuridae, *Taenia* spp., *Parascaris* sp., *Fasciola* sp., and some unknown nematode larvae (Fig. 2). Out of thirty samples, eleven (36.6%) contained helminth's eggs and larvae, including strongyle eggs and larvae in five (16.6%), Ascarid eggs in four (13.3%), *Taenia* spp. eggs in four (13.3%), *Trichuris* spp. eggs in three (10%), *Fasciola* sp. eggs in one (3.3%), and unrecognized nematode larvae in one sample (3.3%) (Table 1). Strongyles were detected in all coprolites except those of birds. Trichuridae was only found in the faeces of the unidentified host. *Taenia* spp. eggs were seen in the unknown and donkey samples and *Fasciola* sp. eggs in donkey faeces. In a total view, helminths were found in 66.6% donkey, 28.5% herbivores, and 26.6% unknown origin coprolites.

**Table 1:** Microscopic & Shotgun sequencing results, botanic origin and dating of fifteen positive palaeofaeces.

Sample number	Microscopic results							Origin	Dating	Shotgun sequencing worm results	
	Ascaris	Strongyle	Trichocephala	Taenia	Fasciola	Unknown larvae	Parascaris			Both NGS and microscope	Just NGS
1	*	*	*					?	Sa	*C. goldi*	
2	*							Bird	Sa	–	–
4		*						Donkey	Sa	–	–
5								Bird	Sa		*F. malakartis*
6		*						Donkey	Sa	–	–
9								Herbivore	Sa		*F. hepatica* *C. goldi* *C. coronatus*
10				*				?	Sa	–	–
12		*						Donkey	Sa	–	–
14								Herbivore	Sa		*C. elegans* *H. contortus*
16	*					*	*	Herbivore	Sa	*P. equorum* *C. elegans*	
24								?	Sa		*C. goldi*
26			*	*				?	Sa		*C. elegans* *C. insignis* *C. nassatus* *H. contortus* *C. goldi*
28		*						Herbivore	Sa	–	–
29			*	*				?	Sa	–	–
30	*			*	*			Donkey	Sa	*T. asiatia* *T. canis*	*S. erinaceieuropaei* *S. mansonoides*

### Helminth metagenomic analysis

**Figure 3:**
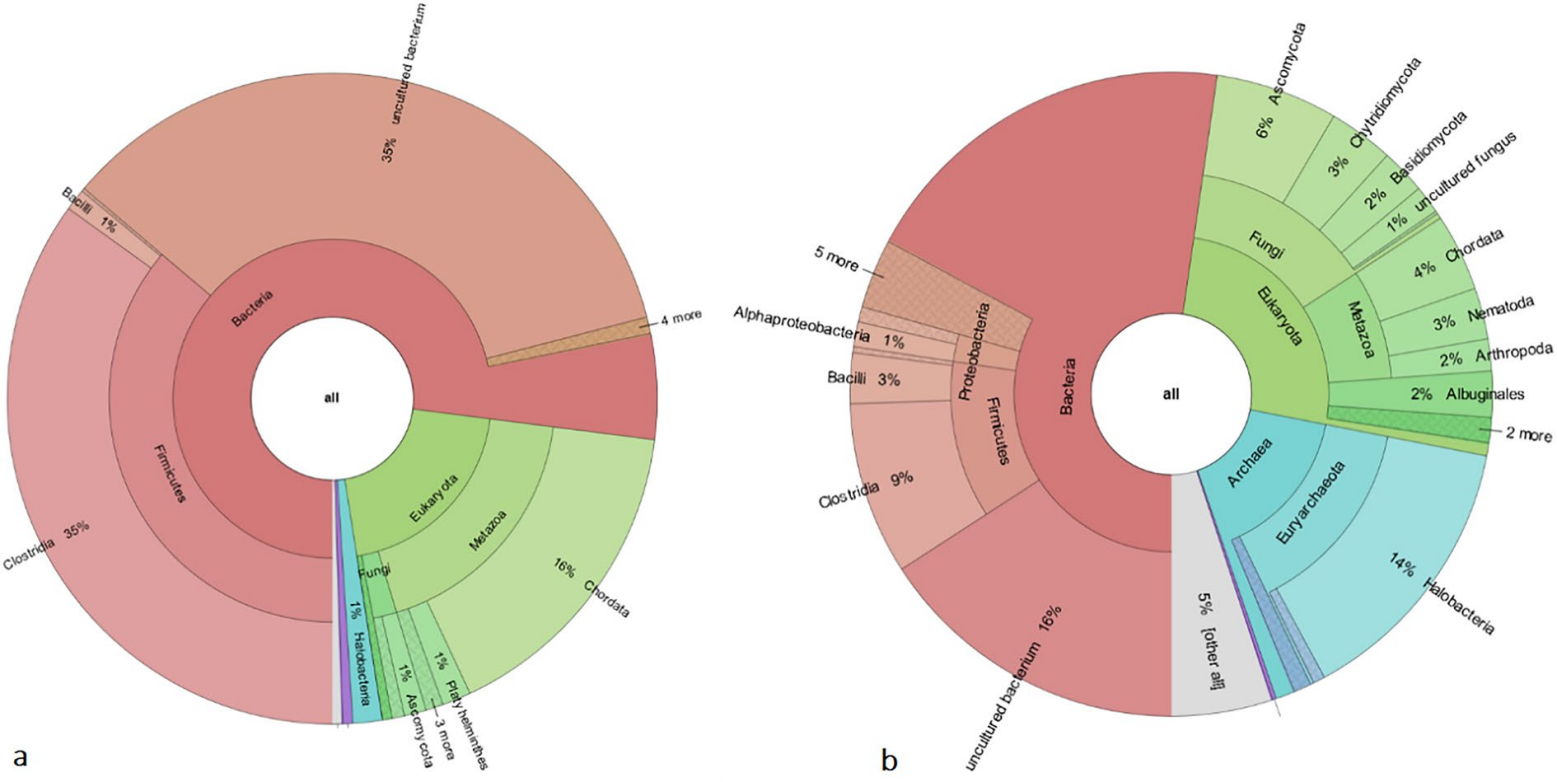
Krona charts of two selected samples: (**a**): sample 9, (**b**): sample 1. Edited by Microsoft paint, version 21 H2, https://www.microsoft.com/en-us/windows.

Various phyla of microorganisms in thirty samples were documented by metagenomics analysis. Out of the studied coprolites, helminth eggs were found in eight samples (Fig. 3, supplementary file result and supplementary file parasitic reads).

### Nematodes

**Figure 4:**
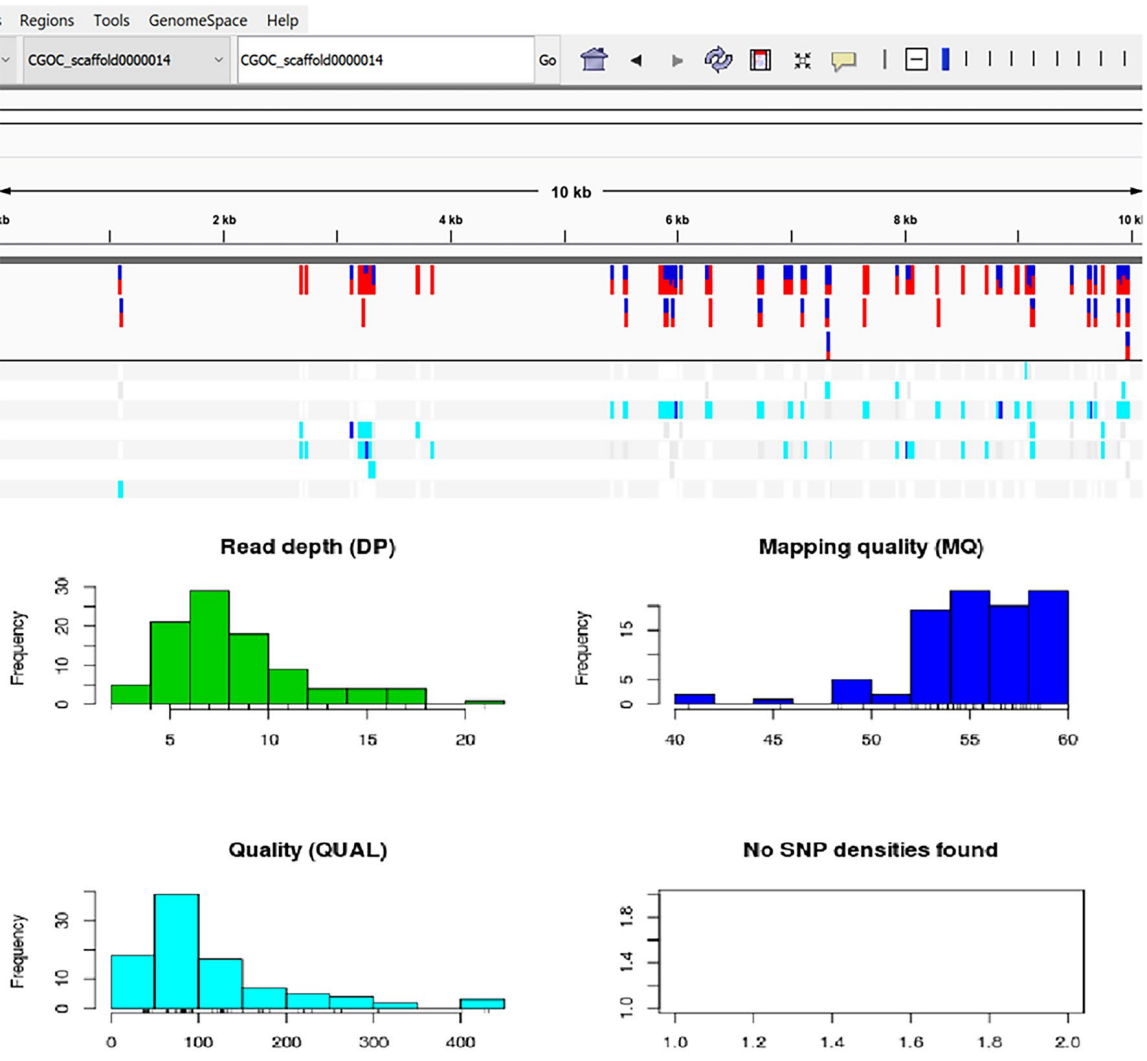
IGV snap shot of *C. goldi* VCF file: IGV view and statistics of. vcf file for reads mapped to *C. goldi* scaffold (CGOC_000014)*.* Edited by Microsoft paint, version 21 H2, https://www.microsoft.com/en-us/windows.

**Figure 5:**
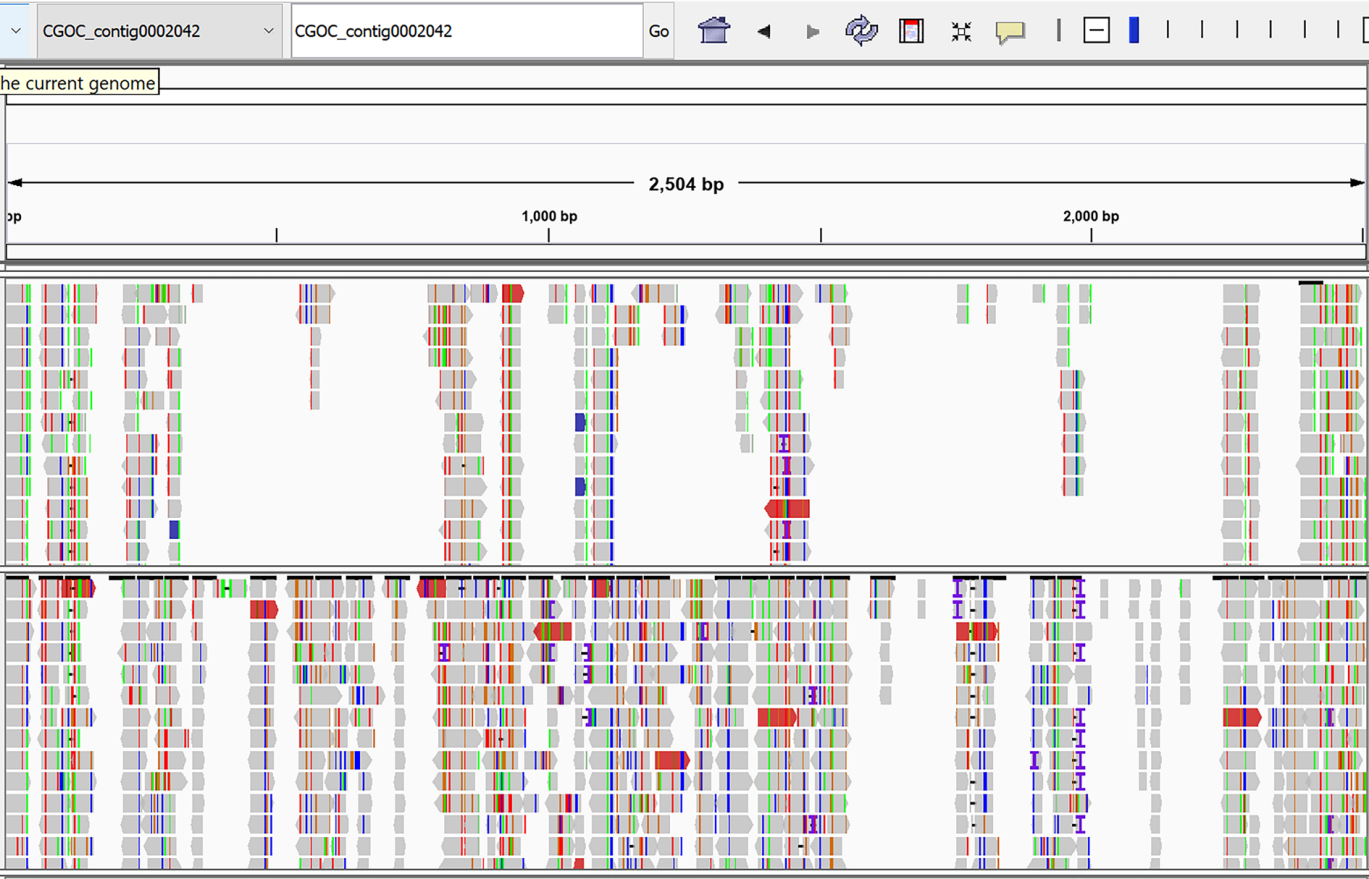
IGV view of damaged and total mapped reads of *C. goldi*: upper track is related to damaged reads. Damage is not presented in whole reads. Edited by Microsoft paint, version 21 H2, https://www.microsoft.com/en-us/windows.

NGS identified DNAs representing *Parascaris equorum* (Supplementary file equorum), four strongyles species, including *Cylicocyclus insignis, Cylicocyclus nassatus, Coronocyclus coronatus,* and *Cylicostephanus goldi*) in the palaeofaeces belonging to herbivores, donkeys, and the unknown hosts. Also, NGS identified *Haemonchus contortus* and *Caenorhabditis elegans* in herbivores and the palaeofaeces of unknown origin, and *Toxocara canis* in a donkey coprolite (Table 1). In *C. goldi* reads, 106,308 indels and 82,299 SNPs were detected with read depth up to fifty-eight (supplementary Fig. s1). The data for CGOC_scaffold0000014 variants are depicted in Fig. 4. The damaged *C*. *goldi* reads*,* separated by the PMDtools (supplementary Fig. s2), showed less frequency than the total aligned reads (Fig. 5). The statistics of total (damaged and undamaged) and reads with substitutions that aligned with the *C. goldi* genome are summarized in the supplementary file total and supplementary file damage. For example, 65,613 reads were mapped to the 2526 nucleotide CGOC_contig0002042, among which 1044 were damaged. According to this file, the uniformity of read distribution to different genomic parts was also apparent.

### Platyhelminthes

Four cestodes, *Fuhrmannetta malakartis, Spirometra erinaceieuropaei, Spirometra mansonoides,* and *T. asiatica* were identified in the palaeofaeces, and *Fasciola hepatica* was the only detected trematode.

In general, *C. goldi* was the most prevalent species (13.3%), followed by *C. elegans* (10%), *H. contortus, C. nassatus* (6.6%), *P. equorum, T. asiatica, T. canis, S. erinaceieuropaei, C. coronatus,* and *S. mansonoides* (3.3%). NGS identified four helminths, *H. contortus, F. malakartis, S. erinaceieuropaei,* and *S. mansonoides*, while the microscopy only identified the relevant genera.

As shown in supplementary Fig. s3 (more details available in the supplementary file asiatica snp), 14 SNPs in ninety *T. asiatica* reads (Supplementary file asiatica reads) exactly matched SNPs related to *T. asiatica* genome. This was also confirmed by the .fsa file (Supplementary file asiatica contig), which exhibited 27 SNPs in common with *T*. *asiatica* (supplementary Fig. s4). In a separate analysis, the 90 reads and a *T. asiatica* contig (TASK contig0004697) were aligned to a *T. saginata* scaffold (Scaffold00355), and while the alignment was less accurate than the previous analysis (less alignment length), the reads shared similar SNPs with the *T. asiatica* genome. (supplementary Fig. s5).

### Origin of specimens

The technical protocol in detecting the faeces origin uses the outer layer of the coprolites, which shows the host intestine cell layer. However, we could not analyze them separately due to the limited amounts of samples. Hence, here we report the vertebrates' DNA alongside microbes (unpublished data) in twenty-five palaeofaeces. The remaining five samples did not contain sufficient data.

**Figure 6:**
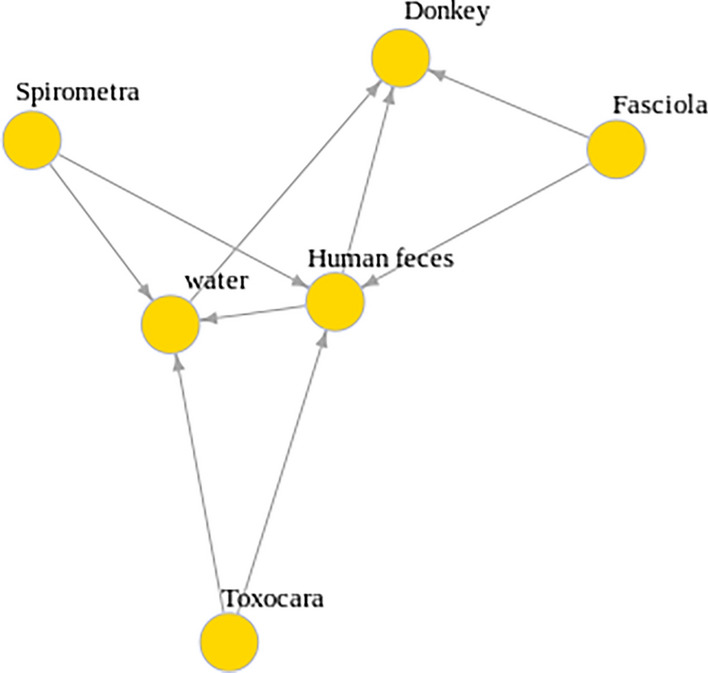
Schematic environmental interactions : Host-Parasite relationships depicted by vcfR.

Technically, finding vertebrates genomic reads in grazing herbivore animals like donkeys cannot attribute the specimen to that animal because they may have consumed various animal excrements, including humans, while grazing. Based on DNA analysis followed by microscopic and botanical findings, the possible interactions and the host-parasite relationship at that time could be imagined (Fig. 6). (Supplementary file origin).

## Discussion

Implementing modern techniques, especially NGS, has provided reliable answers to critical questions raised in many fields, including palaeoparasitology. NGS can detect the biological agents DNA in archaeological remains, including parasites^34^, especially if preserved in suitable conditions, e.g., salt and icy environment^35,36^.

In this study, the intact appearance of the helminth eggs alongside the desired amount of extracted DNAs suggests that the samples were in suitable conditions in this site for ~ 2000 years. Previously, microscopy detected the helminth eggs belonging to *Trichosomoides crassicauda*, *Syphacia* sp., *Trichuris* spp., *Macracanthorhynchus hirudinaceus*, strongles, and Anoplocephalidae of rats, canids, and ungulates in the same archaeological site^37–39^. Moreover, a similar work on mixed soil samples and salt mummies identified the nematodes, i.e., *Trichuris* spp., *Ascaris* sp., *Oxyuris equi, Enterobius vermicularis*, and the Platyhelminthes *Dicrocoelium* sp. and *Taenia* spp^40,41^. Also, microscopical detection of *F. hepatica* eggs in donkey coprolites based on morphometric features at the early stage of the current study is worth mentioning^42^. In this study, microscopy identified the eggs belonging to *Taenia* spp., *Fasciola* sp., *Ascaris* sp*., Trichuris* spp., *Parascaris* sp., strongyles, along with some larvae, and the nematodes were the most prevalent helminths. Strongyles were the most identified helminths by NGS and microscopy. Ingestion of these soil dispersal worms may be to blame for the high prevalence rate of these nematodes in grazing herbivores. The detection of *T. asiatica* DNA raised questions upon this cestode which uses pigs as the intermediate host and humans as the definitive one. The life cycle is completed by ingesting swine's raw or undercooked internal organs harboring the cysticercus larval stage by humans^43,44^. In the present study, the discovery of skeletal remains of a wild boar *Sus scrufa* in the ancient site of Chehrabad can justify the presence of swine-borne parasites in pre-Islamic Iran to some extent^15^. The first report of human infection with Asian tapeworm dates back to 1990s in Asia^45^, followed by human cases from Taiwan, South East Asia, and Russia^46^. The possible occurrence of Asian tapeworm infection in the Sasanian dynasty of Iran in 2000 years ago by tracing the parasite DNA in a donkey's palaeofaeces might suggest that this parasite might have been acquired by humans in a region far from South East Asia. This claim is supported by detecting microbiota DNA of human faeces in the same coprolites most probably ingested by the donkey during grazing on contaminated forage (Supplementary file origin). Moreover, the discovery of *F. malakartis* that was exclusively reported from Egypt^47^, in the palaeofaeces of birds in the current study can be regarded as the first report of this parasite from Iran. Such findings may attract the medical and health experts' attention to consider palaeoparasitological records prior to using the terms" emerging and re-emerging." The route of the Silk Road on the map highlights the possibility of disease transfers between various geographical locations in ancient times. Also, a specific pathogen imported into a new location could have been eliminated due to changes in socio-religious codes after a certain period. These epidemiological views can justify the possible importing of *T. asiatica* into Iran during the Sassanid era through trade from eastern Asia, as well as its disappearance from the country following forbidding commands of pork consumption by Islam in 650 AD. Also, natural interceptive climate change should not be disregarded in addition to these unprecedented socio-cultural changes over time. In our study, strict safety standards were followed to avoid modern contamination. Collecting the coprolites from vertical layers in 10 to 15 m depth, served as a barrier to potential pollutants from the ground surface. Also, no read was obtained in the four reactions that contained only reagents. Moreover, the threat of modern contamination in the sampling phase and the DNA extraction procedure can be neglected, since the main detected parasite i.e., *T. asiatica* is not prevalent in today Iran.

## Supplementary Information

Supplementary Information 1.

Supplementary Information 2.

Supplementary Information 3.

Supplementary Information 4.

Supplementary Information 5.

Supplementary Information 6.

Supplementary Information 7.

Supplementary Information 8.

Supplementary Information 9.

Supplementary Information 10.

Supplementary Information 11.

Supplementary Information 12.

Supplementary Information 13.

Supplementary Information 14.

## Data Availability

The datasets generated and/or analyzed during the current study are accessible in SRA, NCBI repository (https://www.ncbi.nlm.nih.gov/bioproject/PRJNA820553), Bioproject accession number PRJNA820553.
