# Supplementary figures and images for: Genomic palaeoparasitology traced the occurrence of Taenia asiatica in ancient Iran (Sassanid Empire, 2th cent. CE–6th cent. CE)
Source: Sci Rep. 2022 Jul 14;12:12045. doi: 10.1038/s41598-022-10690-2 (PMC9283436; doi:10.1038/s41598-022-10690-2)

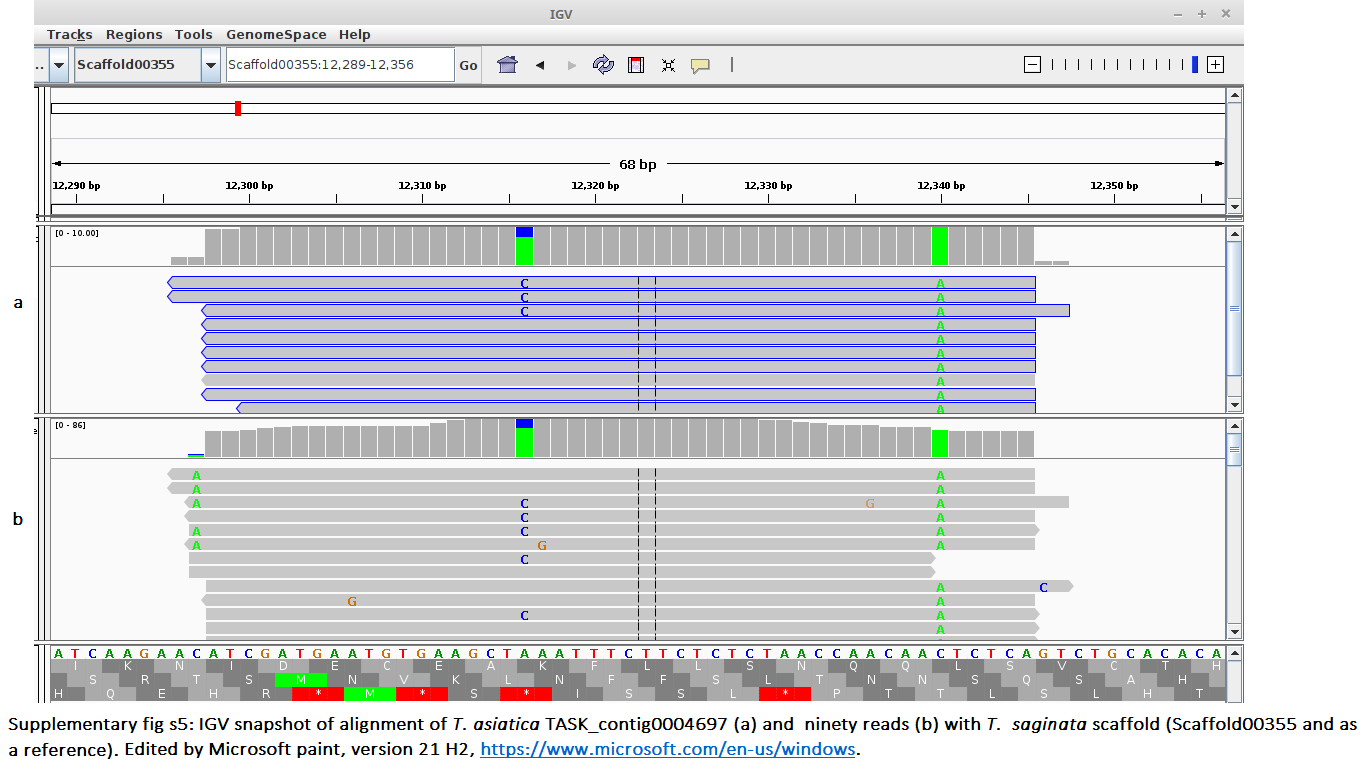

Supplement: Supplementary file 1 — Supplementary Information 1. [file 41598_2022_10690_MOESM1_ESM.png]

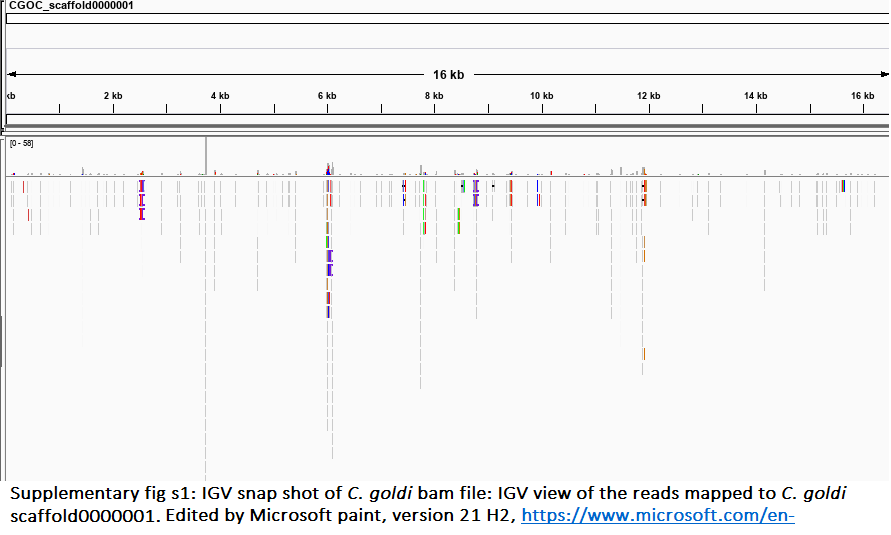

Supplement: Supplementary file 2 — Supplementary Information 2. [file 41598_2022_10690_MOESM2_ESM.png]

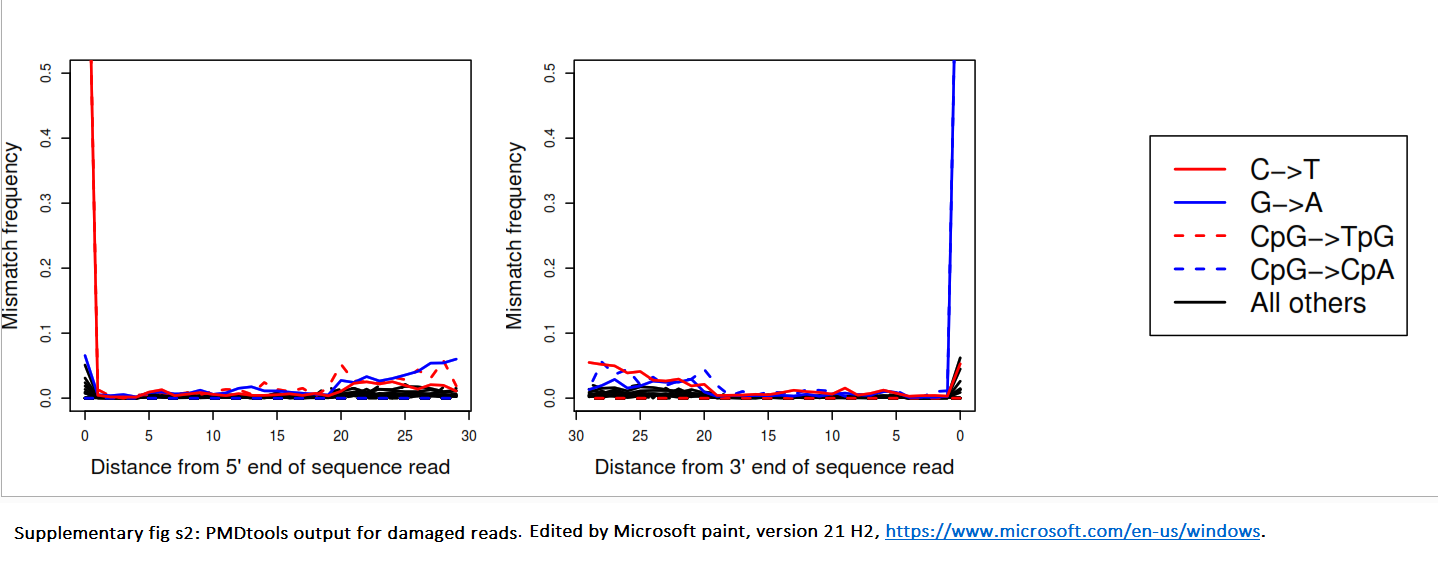

Supplement: Supplementary file 3 — Supplementary Information 3. [file 41598_2022_10690_MOESM3_ESM.png]

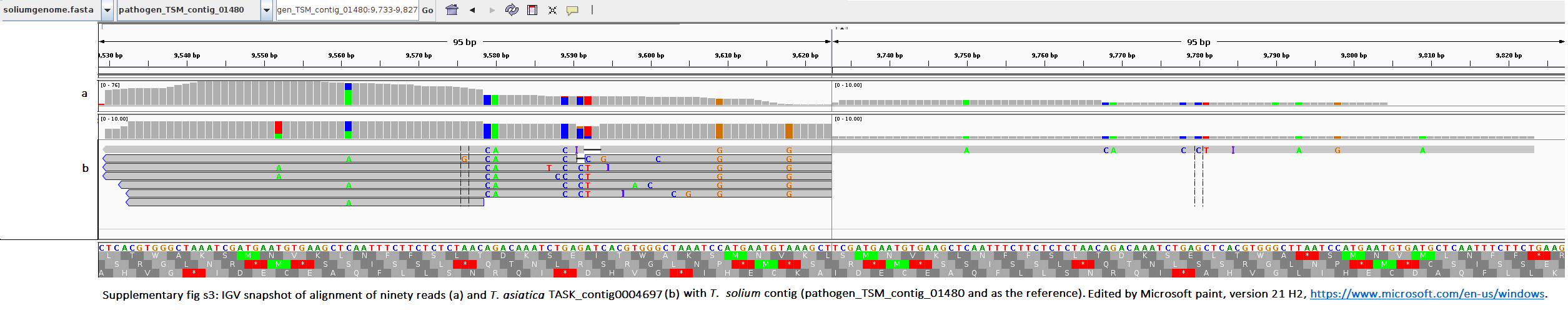

Supplement: Supplementary file 4 — Supplementary Information 4. [file 41598_2022_10690_MOESM4_ESM.png]

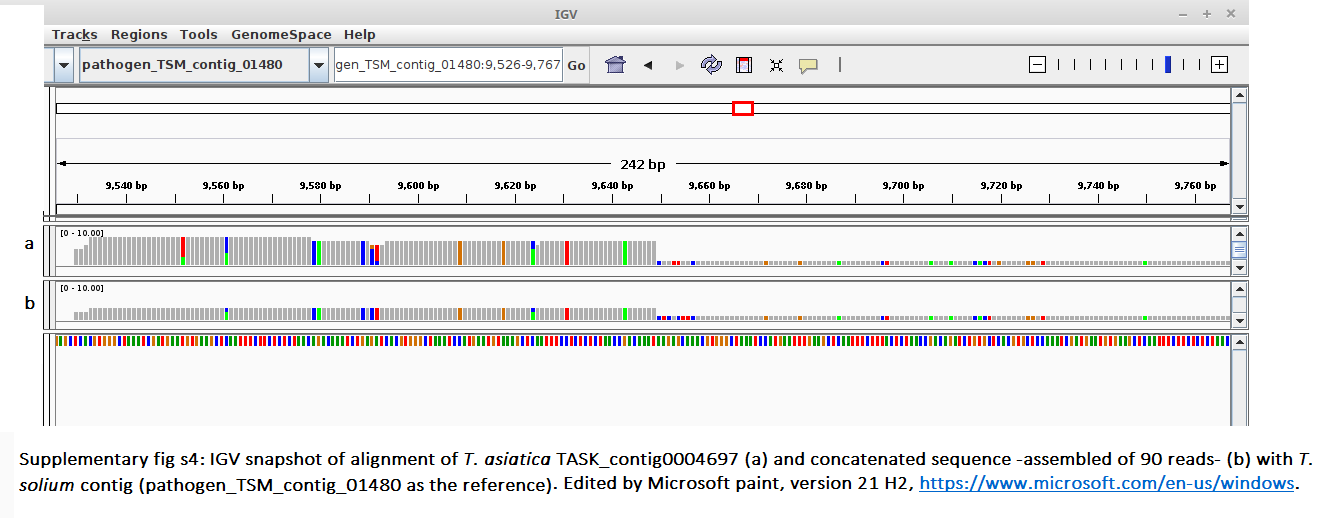

Supplement: Supplementary file 5 — Supplementary Information 5. [file 41598_2022_10690_MOESM5_ESM.png]
