# Supplementary material for: Genomic palaeoparasitology traced the occurrence of Taenia asiatica in ancient Iran (Sassanid Empire, 2th cent. CE–6th cent. CE)
Source: Sci Rep. 2022 Jul 14;12:12045. doi: 10.1038/s41598-022-10690-2 (PMC9283436; doi:10.1038/s41598-022-10690-2)
Supplement: Supplementary file 8 — Supplementary Information 8. [file 41598_2022_10690_MOESM8_ESM.pdf]

Genomic palaeoparasitology traced the occurrence of Taenia asiatica in ancient Iran (Sassanian Empire, 2th cent. CE – 6th cent. CE) by Zeynab Askari, Frank Ruehli, Abigail Bouwman, Vahid Shariati, Saied Reza Naddaf, Domenico Otranto, Santiago Mas-Coma, Mostafa Rezaeian, Nicole Boenke, Thomas Stöllner, Abolfazl Aali, Iraj Mobedi, Gholamreza Mowlavi

Supplementary file asiatica snp: SNP location in *T. asiatica* modern genome, ninety *T. asiatica* reads and concatenated sequence of ninety reads, when “pathogen_TSM_contig_01480:9,524-9,605” in *T. solium* genome is as a reference.
The numbers in third column are the number of reads aligned to that specific position.

SNP position	T. asiatica	The 90 reads	.fsa (concatenate reads)	T. solium
9580	A	C (32)	C	A
9579	A	A(32)	A	G
9589	C	C(26) and T(2)	C	G
9592	T	T(27)	T	A
9609	G	G(20)	G	C
9618	G	G(3)	G	A
9631	T	--	T	C
9643	A		A	G
9650	C		C	A
9651	A		T(unique)	A
9652	G		C(unique)	G
9653	T		A	A
9654	T		C	G
9655	C		T(unique)	C
9656	A		T(unique)	A
9657	C		C	A
9672	G		G	C
9679	G		G	T
9678	A		A	C
9696	C		C	T
9697	T		T	A
9706	A		A	G
9710	A		A	G
9715	C		C	G
9716	A		A	T
9717	C		C	G
9718	T		T	C
9720	G		C	C
9726	G		G	C
9727	G		G	T
9729	T		T	C
9750	A	A(3)	A	C
9768	C	C		A
9769	A	A		G
9778	C	C		G
9780	C	C		G
9781	T	T		C
9793	A	A		T

9798	G	G		C

Here is the table for SNPs which are unique for today's T. asiatica and our .fsa file:

SNP position	T. asiatica	The 90 reads	.fsa (concatenate reads)	T. solium
9651	A		T(unique)	A
9652	G		C(unique)	G
9653	T(unique)		A	A
9655	C		T(unique)	C
9656	A		T(unique)	A
9720	G(unique)		C	C

- 7 SNPs are confirmed by reads, concatenated reads: the high number of reads had same SNPs.
- 18 SNPs are confirmed just by concatenated reads.
- 8 SNPs are confirmed just by reads: one in three reads and the rest in one reads.
- 4 SNPs are common between T. solium and todays T. asiatica.
- 2 SNPs are common between the concatenated reads and T. soium.
